# NDRG1 facilitates lytic replication of Kaposi’s sarcoma-associated herpesvirus by maintaining the stability of the KSHV helicase

**DOI:** 10.1371/journal.ppat.1009645

**Published:** 2021-06-02

**Authors:** Lianghui Dong, Jiazhen Dong, Min Xiang, Ping Lei, Zixian Li, Fang Zhang, Xiaoyi Sun, Danping Niu, Lei Bai, Ke Lan

**Affiliations:** 1 State Key Laboratory of Virology, College of Life Sciences, Wuhan University, Wuhan, China; 2 State Key Laboratory of Cell Biology, CAS Center for Excellence in Molecular Cell Science, Shanghai Institute of Biochemistry and Cell Biology, Chinese Academy of Sciences, University of Chinese Academy of Sciences, Shanghai, China; Wistar Institute, UNITED STATES

## Abstract

The presumed DNA helicase encoded by ORF44 of Kaposi’s sarcoma-associated herpesvirus (KSHV) plays a crucial role in unwinding viral double-stranded DNA and initiating DNA replication during lytic reactivation. However, the regulatory mechanism of KSHV ORF44 has not been fully elucidated. In a previous study, we identified that N-Myc downstream regulated gene 1 (NDRG1), a host scaffold protein, facilitates viral genome replication by interacting with proliferating cell nuclear antigen (PCNA) and the latent viral protein latency-associated nuclear antigen (LANA) during viral latency. In the present study, we further demonstrated that NDRG1 can interact with KSHV ORF44 during viral lytic replication. We also found that the mRNA and protein levels of NDRG1 were significantly increased by KSHV ORF50-encoded replication and transcription activator (RTA). Remarkably, knockdown of NDRG1 greatly decreased the protein level of ORF44 and impaired viral lytic replication. Interestingly, NDRG1 enhanced the stability of ORF44 and inhibited its ubiquitin-proteasome-mediated degradation by reducing the polyubiquitination of the lysine residues at positions 79 and 368 in ORF44. In summary, NDRG1 is a novel binding partner of ORF44 and facilitates viral lytic replication by maintaining the stability of ORF44. This study provides new insight into the mechanisms underlying KSHV lytic replication.

## Author summary

During lytic replication, KSHV ORF44 unwinds viral DNA and initiates DNA replication. Here, we report that the host protein NDRG1, a novel ORF44 binding partner, is significantly upregulated during viral lytic replication and facilitates this process. Mechanistically, NDRG1 can increase the stability of ORF44, impairing the polyubiquitination of the lysine residues at positions 79 and 368 in ORF44, thus inhibiting ubiquitin-proteasome-mediated degradation of ORF44. Our study demonstrates that NDRG1 plays an important role in KSHV lytic replication and may thus constitute a promising therapeutic target for KSHV infection.

## Introduction

Kaposi’s sarcoma-associated herpesvirus (KSHV), also referred to as human herpesvirus 8 and belonging to the human oncogenic γ herpesvirus family, is the etiological agent of several human malignancies, including the endothelial neoplasm Kaposi’s sarcoma (KS) and two B cell lymphoproliferative disorders: primary effusion lymphoma (PEL) and multicentric Castleman’s disease (MCD) [1–3]. Similar to other herpesviruses, the life cycle of KSHV comprises two different phases: latency and lytic replication [4,5]. Extensive evidence has indicated that both the latent and lytic phases of the KSHV life cycle contribute prominently to viral tumorigenesis [6,7]. As a strategy to escape host immune surveillance, KSHV establishes latency for lifelong persistent infection. During latency, KSHV expresses only a few viral genes, and latency-associated nuclear antigen (LANA) tethers the circular extrachromosomal viral episome to the human chromosome for KSHV genome persistence. In addition, LANA plays an essential role in viral DNA replication and transcriptional regulation [8–12]. However, under stimulation by various factors, such as hypoxia, oxidative stress and sodium butyrate, the latency can be disrupted, and lytic reactivation of the virus is induced [13–16]. Moreover, the infectious progeny virus produced by viral lytic replication and constant infection of fresh cells are critical for viral tumorigenicity [17–19]. Therefore, exploring the molecular mechanisms of the viral lytic replication phase is crucial to elucidating viral pathogenesis and may identify potential therapeutic and prophylactic strategies for KSHV-related diseases.

During lytic reactivation, KSHV genome synthesis is essential for the production of mature progeny viruses. The KSHV genome encodes a set of core DNA replication proteins, including ORF6 (ssDNA binding protein), ORF9 (DNA polymerase), ORF40-41 (primase-associated factor), ORF44 (helicase), ORF56 (primase) and ORF59 (polymerase processivity factor) [20]. The presumed KSHV helicase ORF44, one of six core DNA replication proteins, plays an important role in unwinding viral double-stranded DNA and initiating DNA replication, but the regulatory mechanism of the presumed KSHV helicase has not been studied in detail [21]. Our previous study showed that viperin, a cellular antiviral protein, catalyzes methionine-401 oxidation of ORF44 to increase its stability and enzyme activity, with subsequent facilitation of KSHV DNA replication [22]. This finding suggests that ORF44 is elaborately regulated and critical for KSHV lytic replication.

N-Myc downstream regulated gene 1 (NDRG1) is a member of the NDRG family, which consists of four members: NDRG1, NDRG2, NDRG3 and NDRG4. Previously, extensive studies have demonstrated that NDRG1 is a multifunctional protein that is involved in development, cell growth and differentiation, stress responses, myelination, lipid biosynthesis and immunity [23–27]. Recently, the Iizasa group and other groups reported that NDRG1 might be involved in viral infection. A study showed that miRNAs encoded by Epstein-Barr virus (EBV) can downregulate NDRG1, a metastasis suppressor, to promote EBV-mediated epithelial carcinogenesis [28]. Another study showed that NDRG1 suppresses canonical NF-kappa B signaling to facilitate influenza A virus replication [29]. In contrast, a study reported that NDRG1 restricts hepatitis C virus propagation by inhibiting lipid droplet formation and viral assembly [30]. These studies indicate that NDRG1 participates in the viral life cycle and performs diverse functions. In our previous study, we found that NDRG1 is not detectable in B cells and endothelial cells not infected with KSHV, but KSHV infection highly upregulates NDRG1 expression in these cells [31]. Furthermore, we demonstrated that NDRG1 functions as a scaffold protein that forms a complex with LANA and PCNA, helping LANA to load PCNA onto the viral genome and facilitating viral genome replication and episome persistence [31]. These findings suggest that NDRG1 plays an important role in KSHV genome replication during latency, but whether NDRG1 performs other functions in the KSHV life cycle is unclear.

In the present study, we further explored the functions of NDRG1 in KSHV lytic replication. Based on previous data, KSHV ORF44 was identified as a novel NDRG1-binding protein [31]. We further demonstrated that the N-terminal domain of NDRG1 binds to the N-terminal region of ORF44. In addition, we found that the mRNA and protein expression of NDRG1 was highly upregulated by replication and transcription activator (RTA) and the cofactor RBP-Jκ during lytic replication. Remarkably, NDRG1 knockdown dramatically reduced the protein expression level of ORF44 and impaired viral lytic replication. We further showed that NDRG1 inhibits ubiquitin-proteasome-mediated degradation of ORF44 by impairing the polyubiquitination of the lysine (K) residues at positions 79 and 368 in ORF44 to increase its stability. These results demonstrated that NDRG1 plays an essential role in maintaining the stability of ORF44 to facilitate KSHV lytic replication.

## Results

### NDRG1 interacts with KSHV ORF44

In our previous studies, we identified potential interacting proteins of NDRG1 by tandem affinity purification/mass spectrometry (TAP-MS) in the KSHV-positive iSLK.RGB cell line [31]. Among these proteins, 3 KSHV proteins (S1 Table) were enriched in the mass spectrometry data. Based on the number of peptides identified by MS, ORF44 showed high coverage percentage among these viral proteins and was related to DNA replication, indicating that ORF44 might be an important viral binding partner of NDRG1. Based on this result, we speculated that NDRG1 might interact with ORF44, thereby regulating KSHV lytic replication.

**Fig 1:**
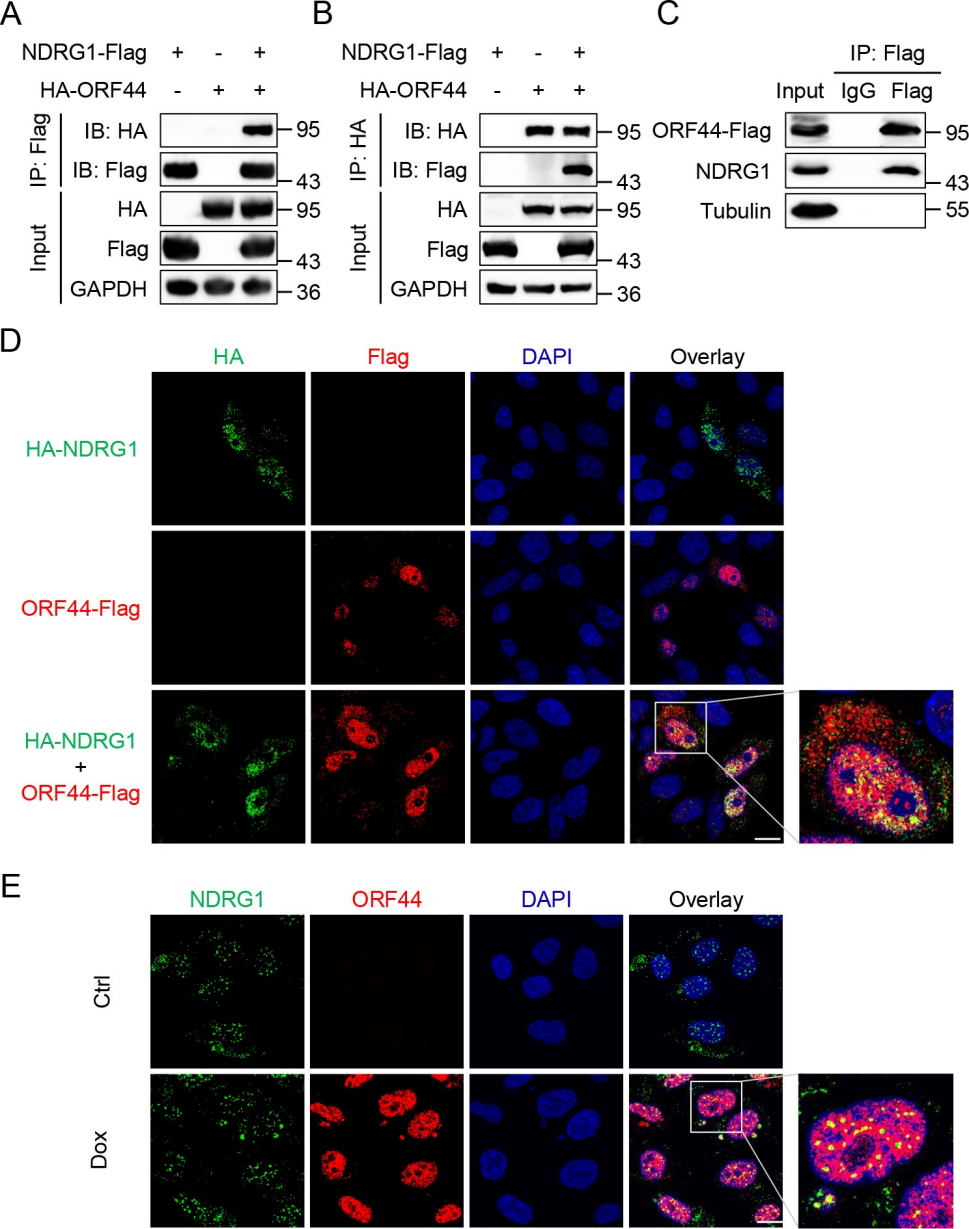
NDRG1 interacts with KSHV ORF44. (A) Immunoprecipitation (IP, with anti-Flag) and immunoblot analysis (with anti-Flag, anti-HA and anti-GAPDH) of HEK293T cells transfected with plasmids encoding Flag-tagged NDRG1 and HA-tagged ORF44 or empty vector for 48 h. (B) Immunoprecipitation (IP, with anti-HA) and immunoblot analysis (with anti-Flag, anti-HA and anti-GAPDH) of HEK293T cells transfected with plasmids encoding Flag-tagged NDRG1 and HA-tagged ORF44 or empty vector for 48 h. (C) Co-immunoprecipitation of endogenous NDRG1 and ORF44 in iSLK-BAC16 KSHV helicase-Flag (GFP deletion) cells. The cells were induced with doxycycline (2 μg/ml) for 48 h, then the cell lysates were immunoprecipitated with anti-Flag rabbit antibody or rabbit IgG control. The immunoprecipitates and input samples were detected by immunoblotting with the indicated antibodies. (D) Colocalization of NDRG1 and ORF44 in HeLa cells. Hela cells were transfected with HA-NDRG1 and ORF44-Flag or empty vector controls. The cells were fixed and probed with anti-HA mouse antibody and anti-Flag rabbit antibody, followed by incubation with goat anti-mouse IgG conjugated with Alexa Fluor 488 (green), goat anti-rabbit IgG conjugated with Alexa Fluor 555 (red), DAPI (blue). Scale bars represent 10 μm. (E) Colocalization of endogenous NDRG1 and endogenous ORF44 in iSLK-BAC16 KSHV helicase-Flag (GFP deletion) cells. iSLK-BAC16 KSHV helicase-Flag (GFP deletion) cells were treated with or without doxycycline for 48 h. The cells were fixed and stained with anti-NDRG1 mouse antibody and anti-Flag rabbit antibody, followed by incubation with the indicated fluorescent secondary antibodies and DAPI. Scale bars represent 10 μm.

To test this hypothesis, we first detected the interaction between NDRG1 and ORF44 by coimmunoprecipitation (Co-IP) assays. As shown in Fig 1A, HEK293T cells were transfected with plasmids encoding Flag-tagged NDRG1 and HA-tagged ORF44 separately or in combination. Forty-eight hours post transfection, the cell lysates were immunoprecipitated with an anti-Flag antibody. ORF44 coimmunoprecipitated with NDRG1 (Fig 1A). Furthermore, the reverse coimmunoprecipitation results showed that NDRG1 coimmunoprecipitated with ORF44 (Fig 1B). To further confirm the endogenous interaction between NDRG1 and ORF44, iSLK-BAC16 KSHV helicase-Flag (GFP deletion) cells, which harbor a KSHV episome expressing C-terminal Flag-tagged ORF44 [22], were induced with doxycycline (Dox) for 48 h to activate KSHV lytic replication. NDRG1 interacted with endogenous ORF44 in KSHV-positive cells during KSHV lytic replication (Fig 1C).

To validate the above results, we performed an immunofluorescence assay (IFA) to explore whether NDRG1 colocalized with ORF44 in the same cellular compartment. HeLa cells were transfected with plasmids encoding HA-tagged NDRG1 and Flag-tagged ORF44 separately or in combination. Forty-eight hours post transfection, the cells were subjected to fixation, permeabilization and blocking and were then incubated with the appropriate primary and secondary antibodies. The results indicated that exogenously transfected NDRG1 and ORF44 were mostly colocalized in the nucleus, but a small amount of these proteins were colocalized in the cytoplasm (Fig 1D). We further showed that endogenous NDRG1 and ORF44 were also mainly colocalized in the nuclear and less colocalized in the cytoplasm of KSHV-positive iSLK-BAC16 ORF44-Flag cells during lytic replication (Fig 1E). Taken together, these results confirmed that KSHV ORF44 is a novel NDRG1-interacting partner.

### Mapping the domains responsible for the interaction between NDRG1 and ORF44

**Fig 2:**
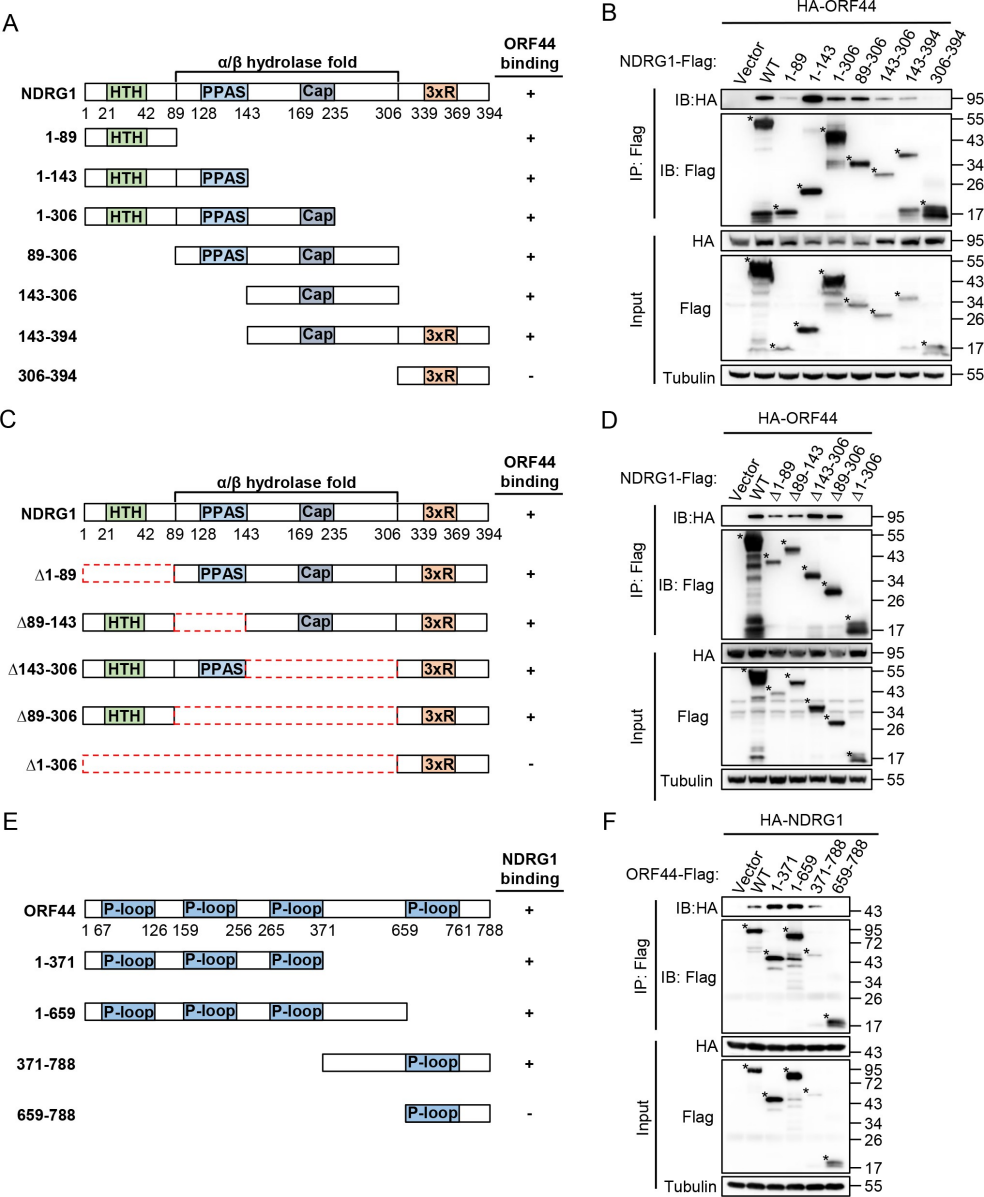
Mapping the domains responsible for the interaction between NDRG1 and ORF44. (A) Schematic diagram of NDRG1 construct and its truncation mutants, including 1–89 (1 aa-89 aa), 1–143 (1 aa-143 aa), 1–306 (1 aa-306 aa), 89–306 (89 aa-306 aa), 143–306 (143 aa-306 aa), 143–394 (143 aa-394 aa), 306–394 (306 aa-394aa). The structural motifs are marked in the NDRG1 protein, which include a helix-turn-helix (HTH) near the N-terminus, an α/β hydrolase fold, a phosphopantetheine attachment site (PPAS) and a cap-like domain and three tandem repeats of GTRSRSHTSE (3xR) in the C-terminus. (B) Defining the ORF44-interacting domain of NDRG1. Immunoprecipitation (with anti-Flag) and immunoblot analysis (with anti-Flag, anti-HA and anti-Tubulin) of HEK293T cells transfected with plasmids encoding HA-tagged ORF44 and Flag-tagged NDRG1 or NDRG1 truncates for 48 h. (C) Schematic diagram of NDRG1 deletion mutants, including Δ1–89, Δ89–143, Δ143–306, Δ89–306, Δ1–306. (D) Immunoprecipitation (with anti-Flag) and immunoblot analysis (with anti-Flag, anti-HA and anti-Tubulin) of HEK293T cells transfected with plasmids encoding HA-tagged ORF44 and Flag-tagged NDRG1 or NDRG1 deletion mutants for 48 h. (E) Schematic diagram of ORF44 construct and its truncation mutants, including 1–371 (1 aa-371 aa), 1–659 (1 aa-659 aa), 371–788 (371 aa-788 aa), 659–788 (659 aa-788 aa). Four P-loop containing nucleoside triphosphate hydrolases superfamily are marked. (F) Defining the NDRG1-interacting domain of ORF44. Immunoprecipitation (with anti-Flag) and immunoblot analysis (with anti-Flag, anti-HA and anti-Tubulin) of HEK293T cells transfected with plasmids encoding HA-tagged NDRG1 and Flag-tagged ORF44 or ORF44 truncates for 48 h.

NDRG1 has some distinctive structural features, including the N-terminal helix-turn-helix (HTH) domain, the α/β hydrolase fold domain, the phosphopantetheine attachment site (PPAS), the cap-like domain, and the C-terminal three tandem repeats of GTRSRSHTSE (3xR) [32,33]. To further delineate the interaction regions in NDRG1 and ORF44, we first constructed a series of Flag-tagged NDRG1 truncation mutants (Fig 2A) to identify the domains in NDRG1 required for its interaction with ORF44. HEK293T cells were transfected with plasmids encoding HA-tagged ORF44 and Flag-tagged NDRG1 or NDRG1 truncation mutants for 48 h. The coimmunoprecipitation results showed that the region of NDRG1 comprising amino acid (aa) residues 1 to 306, which contains the HTH domain and the α/β hydrolase fold domain, is the pivotal region for the interaction between NDRG1 and ORF44 (Fig 2B). Further, we constructed a series of Flag-tagged NDRG1 deletion mutants (Fig 2C) to obtain fine mappings for the interaction. The experimental results showed that only deletion of aa residues 1 to 306 of NDRG1 abolished the interaction of NDRG1 with ORF44, and deletion of aa residues 1 to 89, 89 to 143, 143 to 306 or 89 to 306 of NDRG1 could still interact with ORF44 (Fig 2D), these results indicated that aa residues 1 to 306 of NDRG1 is required for its interaction with ORF44.

Similarly, to explore the domains in ORF44 responsible for its interaction with NDRG1, we predicted the domains of ORF44 using the InterPro database and found that four P-loop-containing nucleoside triphosphate hydrolase domains are the major structural motifs in ORF44. Based on the above finding, we generated a series of Flag-tagged ORF44 truncation mutants (Fig 2E) and performed coimmunoprecipitation assays. The results demonstrated that the N-terminal region (from 1 aa to 659 aa) of ORF44 is responsible for its interaction with NDRG1 (Fig 2F).

### NDRG1 is upregulated by RTA during KSHV lytic replication

**Fig 3:**
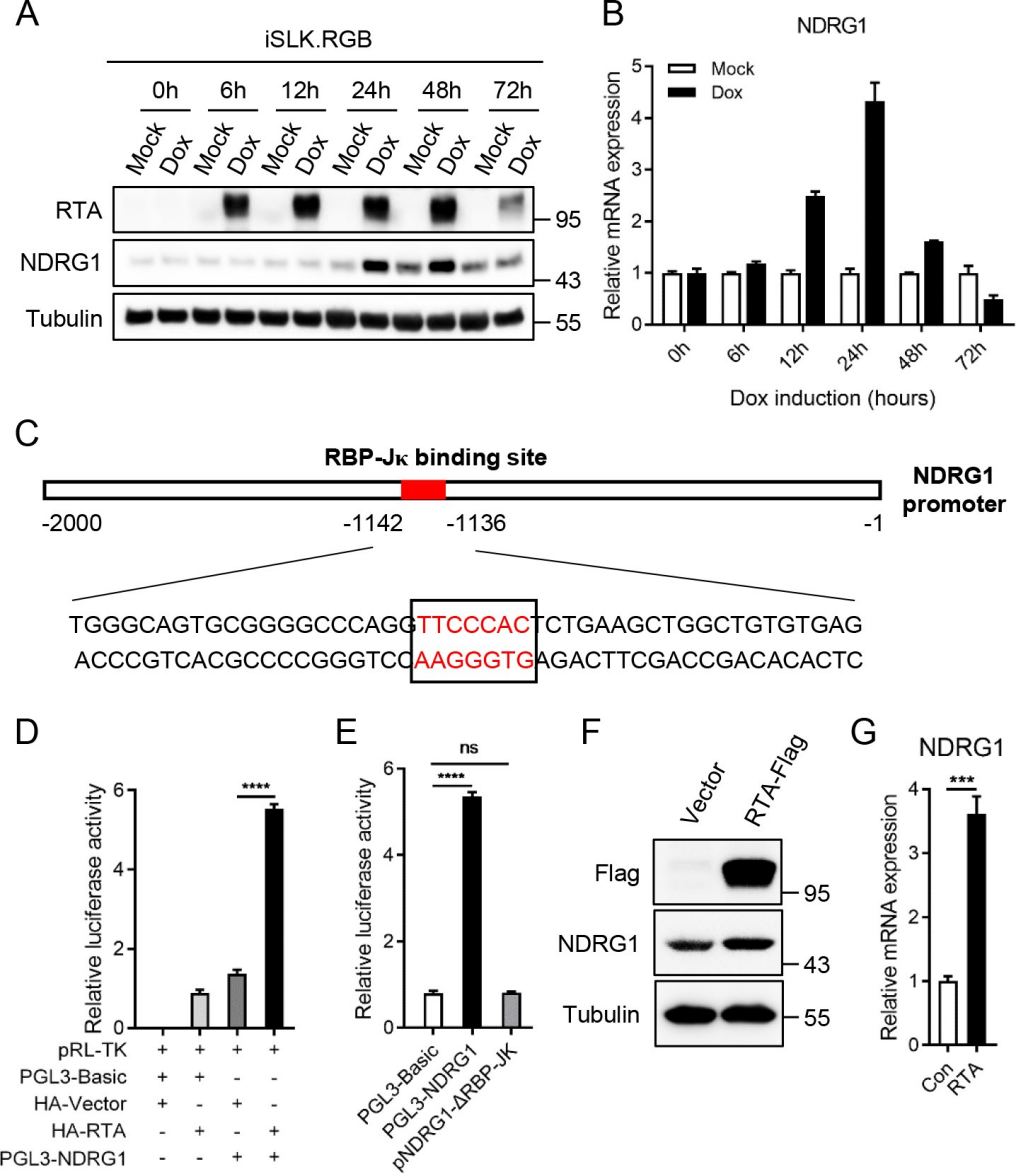
NDRG1 is upregulated by RTA during KSHV lytic replication. (A and B) iSLK.RGB cells were treated with or without doxycycline. The expression kinetics of RTA and NDRG1 at indicated time points were detected by immunoblotting (A) and the mRNA expression of NDRG1 was determined by qPCR analysis (B). (C) Schematic diagram of NDRG1 promoter regions (from -2000 to -1 bp). The RBP-Jκ binding site (from -1142 to -1136 bp) is marked. (D) HEK293T cells were transfected with indicated plasmids for 48 h. Then, the cells were harvested and lysed in cell lysis buffer to detect luciferase activity. (E) HEK293T cells were transfected with indicated plasmids for 48 h and the relative luciferase activity was determined by luciferase assay. (F and G) HEK293T cells were transfected with indicated plasmids. At 48 h post-transfection, the expression of RTA and NDRG1 were detected by immunoblotting (F) and the mRNA expression of NDRG1 was determined by qPCR analysis (G). Data were shown as mean ± SD, n = 3; ns, not significant; ***p < 0.001; ****p <0.0001.

To better explore the function of NDRG1 in KSHV lytic replication, we next examined the expression kinetics of NDRG1 in KSHV-positive iSLK.RGB cells during KSHV lytic reactivation. iSLK.RGB cell line was stably infected with a novel recombinant reporter virus called red-green-blue-BAC16 (RGB-BAC16), which was designed to contain three fluorescent protein expression cassettes—EF1α-monomeric red fluorescent protein 1 (mRFP1), polyadenylated nuclear RNA promoter (pPAN)-enhanced green fluorescent protein (EGFP), and pK8.1-monomeric blue fluorescent protein (tagBFP), markers of latent, immediate early, and late viral gene expression, respectively [34]. Therefore, we can monitor the life cycle of KSHV by visualizing fluorescence, with red fluorescence indicating latent infection and green fluorescence indicating lytic replication. In addition, the iSLK.RGB cell line was transduced with the rtTA (Tet-On) transactivator, which requires doxycycline as a cofactor to induce sufficient KSHV ORF50-encoded RTA expression [35]. Furthermore, RTA, an immediate-early master switch protein, plays a crucial role in triggering the transition from latent viral infection to lytic replication by transactivating downstream viral and cellular gene expression [36,37]. As shown in Fig 3A and 3B, in the context of doxycycline-induced RTA expression, both the protein and mRNA expression of NDRG1 was highly upregulated during KSHV lytic replication (Fig 3A and 3B). To further explore the upregulation mechanism of NDRG1, we first analyzed the sequence of the NDRG1 promoter and found the RBP-Jκ binding site (from -1142 to -1136 bp) in the NDRG1 promoter (Fig 3C). Previous studies have shown that RTA directly interacts with the Notch pathway effector RBP-Jκ (also called CSL), a sequence-specific transcription factor that is constitutively bound to relevant promoter regions at a consensus motif (C/T)GTGGGAA and is indispensable for RTA transactivation activity on cellular and viral promoters [38–41]. Therefore, we speculated whether RTA binds to RBP-Jκ to upregulate NDRG1 expression. To test this hypothesis, we first cloned the NDRG1 promoter region (from -2000 to -1 bp) into the pGL3-Basic luciferase reporter vector. The dual-luciferase reporter assay results showed that RTA was crucial for transactivation of the NDRG1 promoter (Fig 3D). Then, we deleted the RBP-Jκ binding site in the NDRG1 promoter, and the experimental results showed that the NDRG1 promoter was not activated by RTA, indicating that RBP-Jκ, participates as a cofactor in the transactivation of NDRG1 by RTA (Fig 3E). Similarly, HEK293T cells were transiently transfected with Flag-tagged RTA, and both the protein and mRNA expression of NDRG1 was upregulated (Fig 3F and 3G). To further support our hypothesis, we knocked down RTA in iSLK.RGB cells with two RTA-specific siRNAs (S1A Fig). The results of immunoblotting and qPCR analysis showed that RTA depletion significantly reduced the protein and mRNA expression levels of NDRG1 during KSHV lytic reactivation (S1B Fig). These results indicated that the transcription factor RTA and the cofactor RBP-Jκ act on the NDRG1 promoter to upregulate NDRG1 expression during KSHV lytic replication.

### Knockdown of endogenous NDRG1 impairs KSHV lytic replication

**Fig 4:**
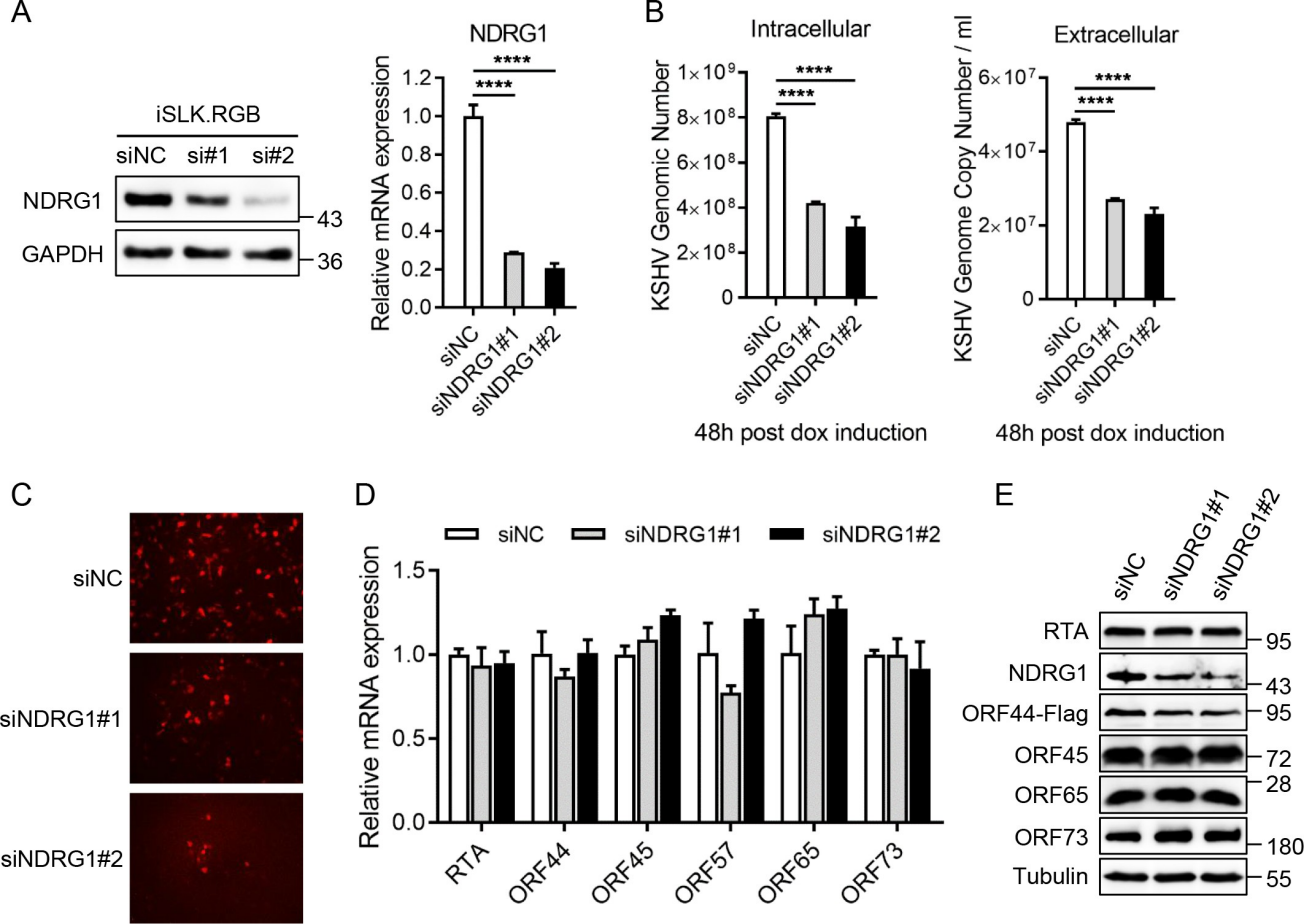
Knockdown of endogenous NDRG1 impairs KSHV lytic replication. (A) iSLK.RGB cells were transfected with siRNA as indicated. At 24 h post-transfection, the cells were treated with doxycycline for 48 h. The knockdown efficiency of NDRG1 was determined by immunoblotting (left panel) and qPCR analysis (right panel). (B) Intracellular viral genomic DNA (left panel) and extracellular virion DNA (right panel) were extracted from induced iSLK.RGB cells or the cell supernatants. Then, the KSHV genomic DNA copy numbers were quantified by qPCR analysis. (C) HEK293T cells were incubated with the indicated cells supernatants (1ml) at 48 h post dox induction. At 24 h post-infection, the virus infection rate of HEK293T cells was detected by fluorescence microscopy according to RFP signal intensity. (D) The transcription levels of several KSHV genes at 48 h post dox induction were determined by qPCR analysis. (E) iSLK-BAC16 KSHV helicase-Flag cells were transfected with siRNA as indicated. At 24 h post-transfection, the cells were induced with doxycycline for 48 h. The protein expression of NDRG1, RTA, ORF44-Flag, ORF45, ORF65 and ORF73 were detected by immunoblotting. Data were shown as mean ± SD, n = 3; ****p <0.0001.

To explore the function of NDRG1 in KSHV lytic replication, we knocked down endogenous NDRG1 in iSLK.RGB cells with two specific siRNAs (siNDRG1#1 and siNDRG1#2) and a negative control siRNA (siNC) and induced the cells with doxycycline. As shown in Fig 4A, the protein and mRNA expression levels of NDRG1 were greatly reduced by the indicated siRNAs (Fig 4A). Indeed, knockdown of endogenous NDRG1 dramatically decreased the production of KSHV virions and KSHV genome replication (Fig 4B). Furthermore, progeny virions in equal volumes of cell culture supernatants were used to infect HEK293T cells, and the red fluorescence intensity in the two NDRG1 knockdown groups was observably decreased compared to that in the control group (Fig 4C). In addition, knockdown of NDRG1 did not affect the transcription levels of viral genes (Fig 4D) or the protein expression levels of viral RTA, ORF45, ORF65 and ORF73. However, the protein expression level of ORF44 in the NDRG1 knockdown groups was reduced compared to that in the control group (Fig 4E).

### Knockout of endogenous NDRG1 impairs KSHV lytic replication

**Fig 5:**
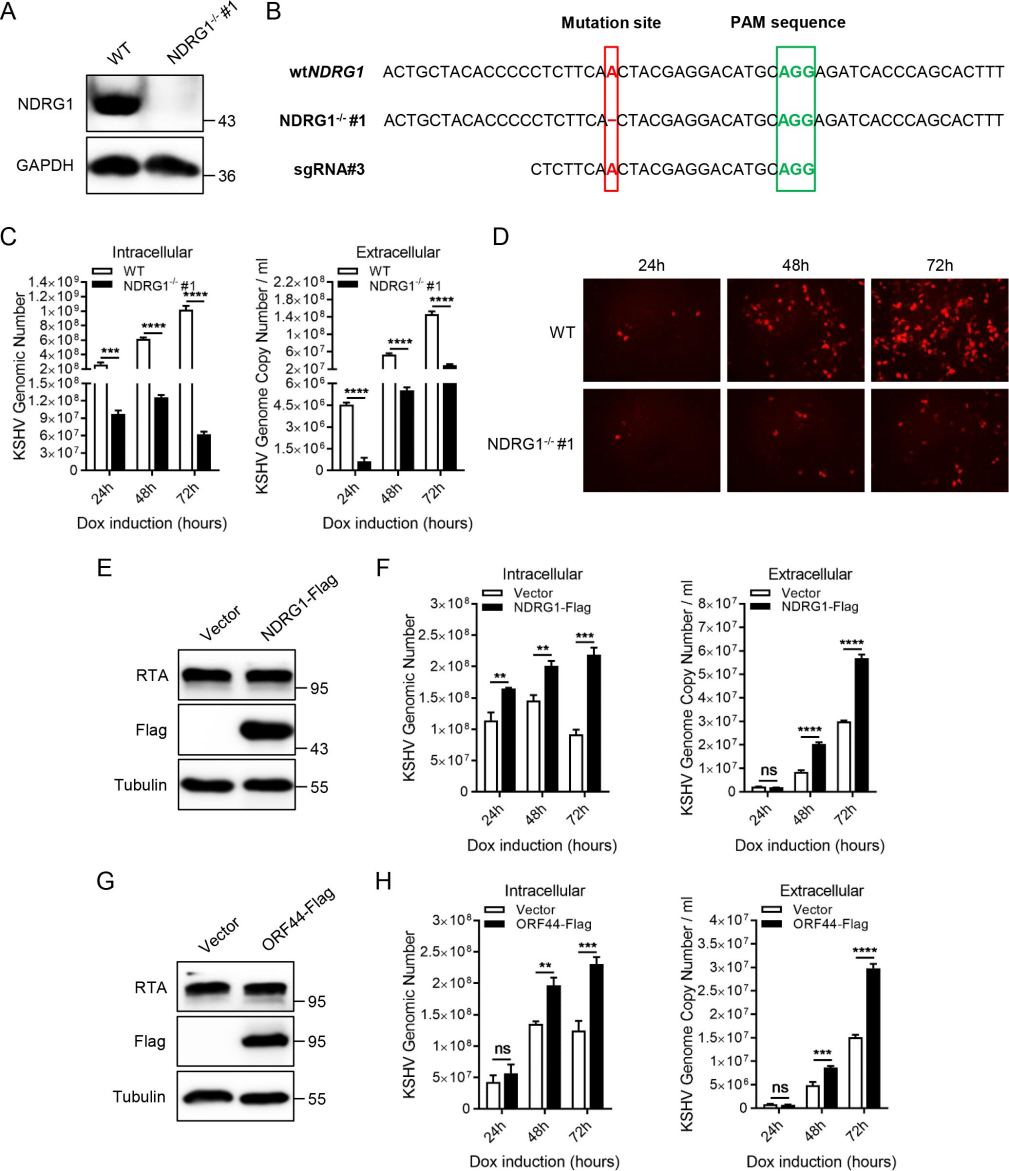
Knockout of endogenous NDRG1 impairs viral lytic replication. (A and B) NDRG1-deficient iSLK.RGB cell line was constructed through the lentiviral CRISPR/Cas9 system. The knockout efficiency of NDRG1 was confirmed by immunoblotting and Sanger sequencing. (C and D) Wild-type iSLK.RGB cells (WT) and NDRG1-deficient iSLK.RGB cells (NDRG1^-/-^) were treated with doxycycline. Intracellular viral genomic DNA and extracellular virion DNA were extracted from the induced cells or cells supernatants. The KSHV genomic DNA copy numbers at indicated time points were detected by qPCR analysis (C). The culture supernatants (1ml) collected from the indicated cells at indicated post dox induction time points were incubated with HEK293T cells. At 24 h post-infection, the virus infection rate of HEK293T cells was detected by fluorescence microscopy according to RFP signal intensity (D). (E and F) NDRG1-deficient iSLK.RGB cells were infected with the indicated lentivirus, after 24 h, the cells were treated with doxycycline and the cells and supernatants were collected at indicated time points. The protein expression of RTA and NDRG1-Flag were detected by immunoblotting (E) and intracellular and extracellular KSHV genomic DNA copy numbers at indicated time points were determined by qPCR analysis (F). (G and H) NDRG1-deficient iSLK.RGB cells were infected with the indicated lentivirus, at 24 h post-infection, the cells were induced by doxycycline, then the cells and supernatants were collected at indicated time points. The protein expression of RTA and ORF44-Flag were confirmed by immunoblotting (G) and intracellular and extracellular KSHV genomic DNA copy numbers at indicated time points were detected by qPCR analysis (H). Data were shown as mean ± SD, n = 3; ns, not significant; **p < 0.01; ***p < 0.001; ****p <0.0001.

To further verify the above results, an NDRG1-deficient iSLK.RGB cell line was generated by a lentiviral CRISPR/Cas9 system. NDRG1 knockout was confirmed by immunoblotting and Sanger sequencing (Fig 5A and 5B). As expected, the experimental results showed that NDRG1 deletion dramatically reduced both the extracellular and intracellular viral genome copy numbers (Fig 5C). Similarly, we tested other individual NDRG1-deficient iSLK.RGB cell clones and found that these cells exhibited consistent viral phenotypes (S2 Fig). In addition, progeny virions in equal volumes of cell culture supernatants collected at different time points during doxycycline induction were incubated with HEK293T cells, and the red fluorescence intensity in the NDRG1 knockout groups was significantly decreased (Fig 5D). To further confirm the role of NDRG1, NDRG1-deficient iSLK.RGB cells were infected with the lentivirus comprising Flag-tagged NDRG1 plasmid to restore NDRG1 expression. At 24 h post-infection, the cells were induced by doxycycline, then the cells and supernatants were collected at indicated time points (Fig 5E). The experimental results showed that both the intracellular and extracellular viral genome copy numbers were partly increased compared to those in the control group (Fig 5F). Moreover, the above results indicated that NDRG1 depletion greatly reduced the protein expression of ORF44. Thus, we sought to determine whether restoration of ORF44 expression in NDRG1-deficient iSLK.RGB cells promotes KSHV lytic replication. Similarly, the lentivirus comprising Flag-tagged ORF44 plasmid infected NDRG1-deficient iSLK.RGB cells and restoration of ORF44 expression dramatically increased both intracellular and extracellular viral genome copy numbers (Fig 5G and 5H). In general, these results demonstrated that the host protein NDRG1 is essential for KSHV lytic replication, indicating that NDRG1 enhances the protein expression of ORF44 to facilitate viral lytic replication.

### NDRG1 increases the stability of ORF44 by inhibiting its ubiquitin-proteasome-mediated degradation

**Fig 6:**
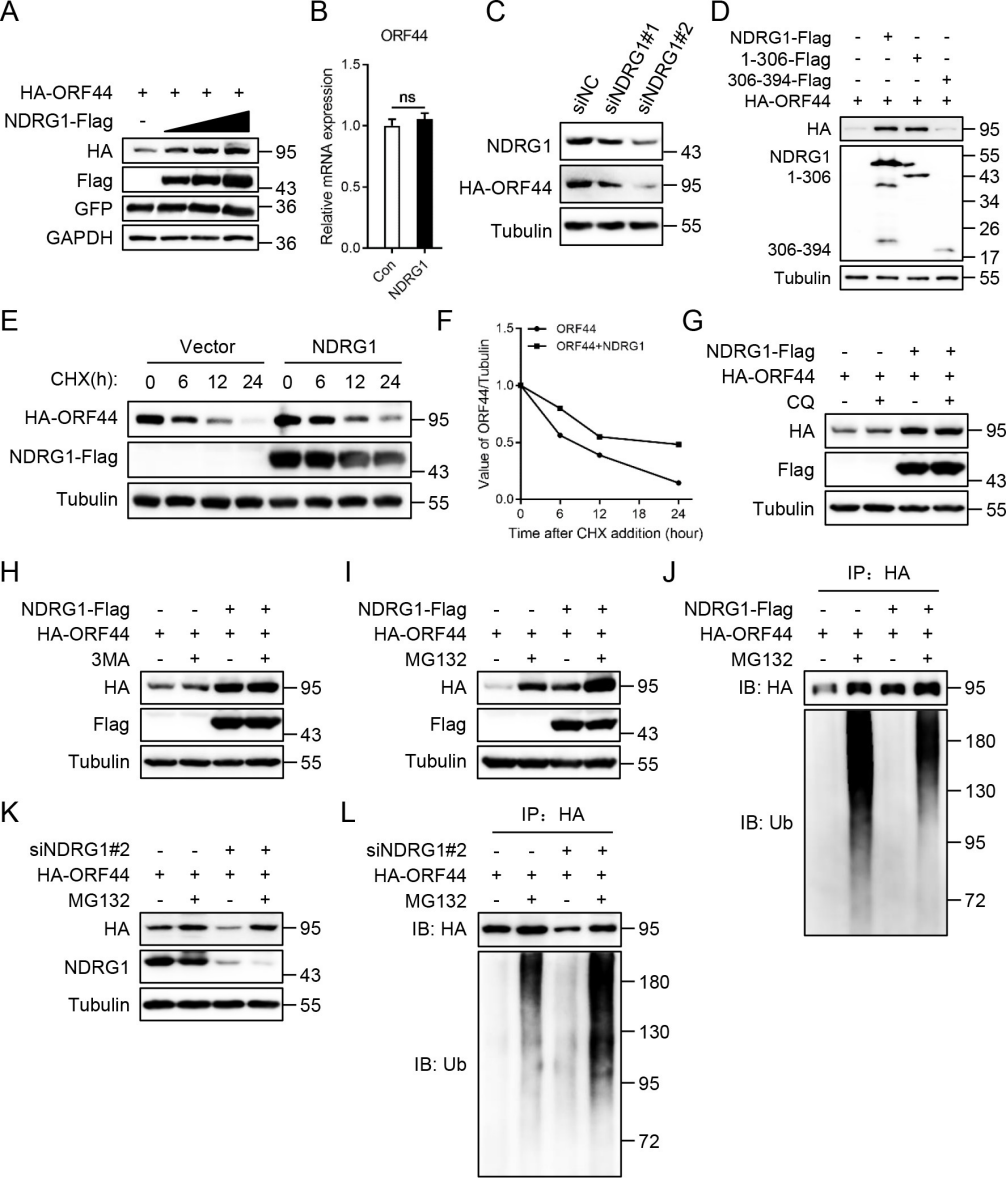
NDRG1 increases the stability of ORF44 by inhibiting its ubiquitin-proteasome-mediated degradation. (A) HEK293T cells were transfected with plasmids encoding HA-tagged ORF44 (0.5 μg) and Flag-tagged NDRG1 (0, 0.5, 1, 2 μg) or empty vector for 48 h. The protein expression of ORF44 and NDRG1 were detected by immunoblotting. (B) HEK293T cells were transfected with indicated plasmids for 48 h. The mRNA expression of ORF44 was determined by qPCR analysis. (C) HEK293T cells were transfected with siRNA as indicated for 12 h. Then, the cells were transfected with plasmid encoding HA-tagged ORF44 for 36 h. The protein expression of NDRG1 and ORF44 were determined by immunoblotting. (D) HEK293T cells were transfected with plasmids encoding HA-tagged ORF44 and Flag-tagged NDRG1 or NDRG1 truncates for 48 h. The protein expression of ORF44 was detected by immunoblotting. (E) HEK293T cells were transfected with plasmids encoding HA-tagged ORF44 and Flag-tagged NDRG1 or empty vector for 36 h, then the cells were treated with cycloheximide (CHX) (20μM) for 12 h. The protein expression of ORF44 and NDRG1 at indicated time points were analyzed by immunoblotting. (F) Values were calculated from the experiments shown in panel E. Signal intensities first were normalized to the corresponding tubulin and then normalized to the signal of each group at 0 h. (G-I) HEK293T cells were transfected with plasmids encoding HA-tagged ORF44 and Flag-tagged NDRG1 or empty vector for 36 h. The cells were treated with or without chloroquine (CQ) (50 μM) (G), 3MA (0.5 mM) (H) or MG132 (20 μM) (I) for 12 h. Then, the cells were lysed and the protein expression of ORF44 and NDRG1 were analyzed by immunoblotting. (J) The same cell lysates in panel I were immunoprecipitated with anti-HA antibody. The immunoprecipitates were analysed by immunoblotting with the indicated antibodies. (K) HEK293T cells were transfected with siRNA as indicated for 12 h. Then, the cells were transfected with plasmid encoding HA-tagged ORF44 and treated with MG132 for 12 h. The cells were lysed for immunoblotting with the indicated antibodies. (L) The same cell lysates in panel K were immunoprecipitated with anti-HA antibody. The immunoprecipitates were detected by immunoblotting with the indicated antibodies. Data were shown as mean ± SD, n = 3; ns, not significant.

The above data demonstrated that NDRG1 interacts with ORF44 and that silencing of NDRG1 reduces the protein level of ORF44 only during viral lytic replication. Therefore, we hypothesized that NDRG1 affects the stability of ORF44. To verify this hypothesis, HEK293T cells were cotransfected with a gradually increasing amount of Flag-tagged NDRG1 plasmids and a constant amount of HA-tagged ORF44 plasmids. The immunoblotting results showed that NDRG1 enhanced ORF44 protein expression in a dose-dependent manner (Fig 6A) but did not affect the mRNA expression of ORF44 (Fig 6B). On the other hand, HEK293T cells were transiently transfected with HA-tagged ORF44 after endogenous NDRG1 knockdown, and the immunoblotting results indicated that knockdown of NDRG1 greatly reduced the protein expression level of ORF44 (Fig 6C). Moreover, similar to the full-length NDRG1 protein, the NDRG1 truncation mutant N306 upregulated ORF44 protein expression (Fig 6D). To further determine the role of NDRG1 in regulating ORF44 protein expression, HA-tagged ORF44 was transfected into HEK293T cells with or without Flag-tagged NDRG1 for 36 h, and the cells were then treated with the protein synthesis inhibitor cycloheximide (CHX) for 12 h. The experimental results indicated that coexpression of NDRG1 significantly prolonged the half-life of ORF44 (Fig 6E and 6F). Taken together, these results demonstrated that NDRG1 increases the stability of ORF44.

Our previous study showed that Viperin directly interacts with ORF44 to catalyze methionine-401 oxidation of ORF44 and increase its stability and enzyme activity [22]. Therefore, we speculated whether Viperin is required for NDRG1 to increase the stability of ORF44. To prove this hypothesis, we knocked down Viperin in HEK293T cells with two Viperin-specific siRNAs (S3A Fig), then the cells were transiently transfected with plasmids encoding HA-tagged ORF44 and Flag-tagged NDRG1 or empty vector for 48 h. The immunoblotting results showed that knockdown of Viperin did not affect the upregulation of ORF44 expression by NDRG1(S3B Fig), indicating that NDRG1 increases ORF44 stability independent of Viperin.

Many studies have shown that both the autophagy-lysosome pathway and ubiquitin-proteasome pathway are major intracellular degradation systems in eukaryotes [42–45]. Therefore, we speculated that NDRG1 probably maintains the stability of ORF44 by suppressing certain protein degradation pathways. To verify this hypothesis, the plasmid encoding HA-tagged ORF44 was transfected into HEK293T cells with or without Flag-tagged NDRG1 for 36 h, and the cells were then treated separately with the lysosomal inhibitor chloroquine (CQ), autophagy inhibitor 3MA or proteasome inhibitor MG132 for 12 h. The immunoblotting results showed that the proteasome inhibitor MG132 but not the lysosomal inhibitor chloroquine or autophagy inhibitor 3-MA dramatically rescued ORF44 degradation (Fig 6G, 6H and 6I). In the absence of ectopic NDRG1 expression, the protein abundance of ORF44 was markedly increased in the cells exposed to MG132 (Fig 6I, lanes 1 and 2). However, in the context of ectopic NDRG1 expression, the protein expression level of ORF44 was significantly enhanced independent of treatment with MG132 (Fig 6I, lanes 3 and 4). To further explore the effect of NDRG1 on ORF44 ubiquitination, the treated cells were lysed and immunoprecipitated with an anti-HA antibody prior to immunoblotting with an anti-ubiquitin antibody. As expected, in cells treated with MG132, a high molecular weight polyubiquitin-ORF44 smear was obvious in cells without ectopic NDRG1 expression (Fig 6J, lane 2), but the polyubiquitination level of ORF44 was dramatically decreased in cells overexpressing NDRG1 (Fig 6J, lane 4). In contrast, knockdown of NDRG1 expression dramatically decreased the protein expression level of ORF44 (Fig 6K, lane 3), but MG132 significantly rescued the degradation of ORF44 (Fig 6K, lane 4). Similarly, knockdown of NDRG1 obviously enhanced the polyubiquitination level of ORF44 compared to that in negative control cells (Fig 6L, lanes 2 and 4). In general, these results demonstrated that NDRG1 impairs the polyubiquitination of ORF44 to inhibit its ubiquitin-proteasome-mediated degradation.

### NDRG1 impairs the polyubiquitination of the lysine residues at positions 79 and 368 in ORF44

**Fig 7:**
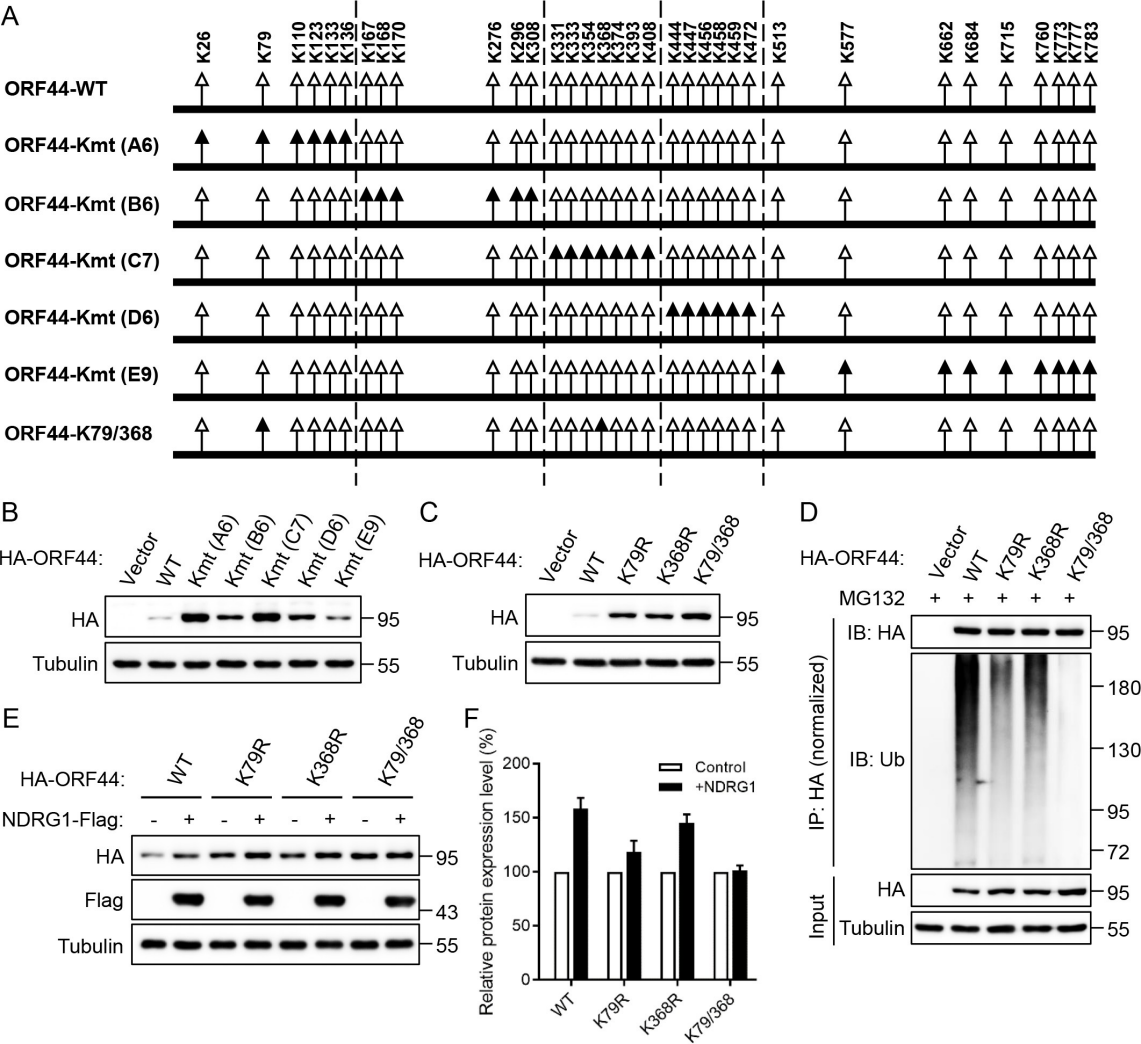
NDRG1 impairs the polyubiquitination of the lysine residues at positions 79 and 368 in ORF44. (A) Schematic diagram of ORF44 wild-type and ORF44 lysine mutants. The positions of 34 lysine residues in ORF44 are marked in the diagram (triangles). In addition, the black triangles represent the replacements of lysine (K) with arginine (R) in ORF44. (B) HEK293T cells were transfected with the multiple-lysine mutants of ORF44 for 48 h, and the protein expression of these mutants were detected by immunoblotting. (C) HEK293T cells were transfected with single- or double-lysine mutants at K79 and K368 of ORF44 for 48 h, the protein abundance of these mutants were demonstrated with immunoblotting. (D) HEK293T cells were transfected with the indicated plasmids for 36h, followed by treating with MG132 for 12 h. Then, the cells were lysed and immunoprecipitated with anti-HA antibody. The immunoprecipitates were analysed by immunoblotting with the indicated antibodies. (E) The indicated plasmids were transfected into HEK293T cells with or without NDRG1 for 48 h, then the protein expression of these mutants were detected with immunoblotting. (F) Relative protein levels of ORF44 mutants in the presence or absence of NDRG1. The bar chart summarizes the band intensities data of ORF44 mutants from three independent experimental replicates with a standard deviation.

Our analysis of the ORF44 protein sequence showed that ORF44 contains 34 lysine residues (Fig 7A). To identify the critical lysine residues required for regulating the stability of ORF44, ORF44 was divided into five clusters based on the positions of the lysine residues, namely, A6, B6, C7, D6 and E9 (S4A Fig). Then, all lysine residues in each cluster were replaced with arginine residues to generate five mutants: Kmt (A6), Kmt (B6), Kmt (C7), Kmt (D6) and Kmt (E9) (Fig 7A). These ORF44 mutants were transfected into HEK293T cells, and the immunoblotting results showed that the protein abundances of ORF44-Kmt (A6) and ORF44-Kmt (C7) were significantly higher than those of ORF44-wild type (WT) and the other ORF44 mutants (Fig 7B). These results indicated that the lysine residues from K26 to K136 and K331 to K408 play a key role in controlling the stability of ORF44. To further search for the crucial ubiquitination sites, we generated a series of ORF44 single lysine mutants and transfected them into HEK293T cells. Immunoblot analysis showed that the single mutation of K79 or K368 in ORF44 most observably enhanced the protein stability of ORF44 compared to that of ORF44-WT and the other mutants (S4 Fig). In addition, double mutation of K79 and K368 in ORF44 further increased the protein abundance of ORF44 (Fig 7C). As expected, the single mutation of K79 or K368 in ORF44 dramatically decreased the polyubiquitination level of ORF44, and double mutation of K79 and K368 in ORF44 nearly abolished its polyubiquitination (Fig 7D). To explore whether NDRG1 affects the ubiquitination of K79 and K368 in ORF44 to increase ORF44 protein abundance, ORF44-WT and the indicated mutants were transfected into HEK293T cells with or without NDRG1. The immunoblotting results indicated that NDRG1 slightly enhanced the protein stability of ORF44-Kmt (K79R) and ORF44-Kmt (K368R) but did not affect the protein abundance of ORF44-Kmt (K79/368) (Fig 7E and 7F). Taken together, these results demonstrated that NDRG1 impairs the polyubiquitination of residues K79 and K368 in ORF44 to increase its protein stability.

## Discussion

**Fig 8:**
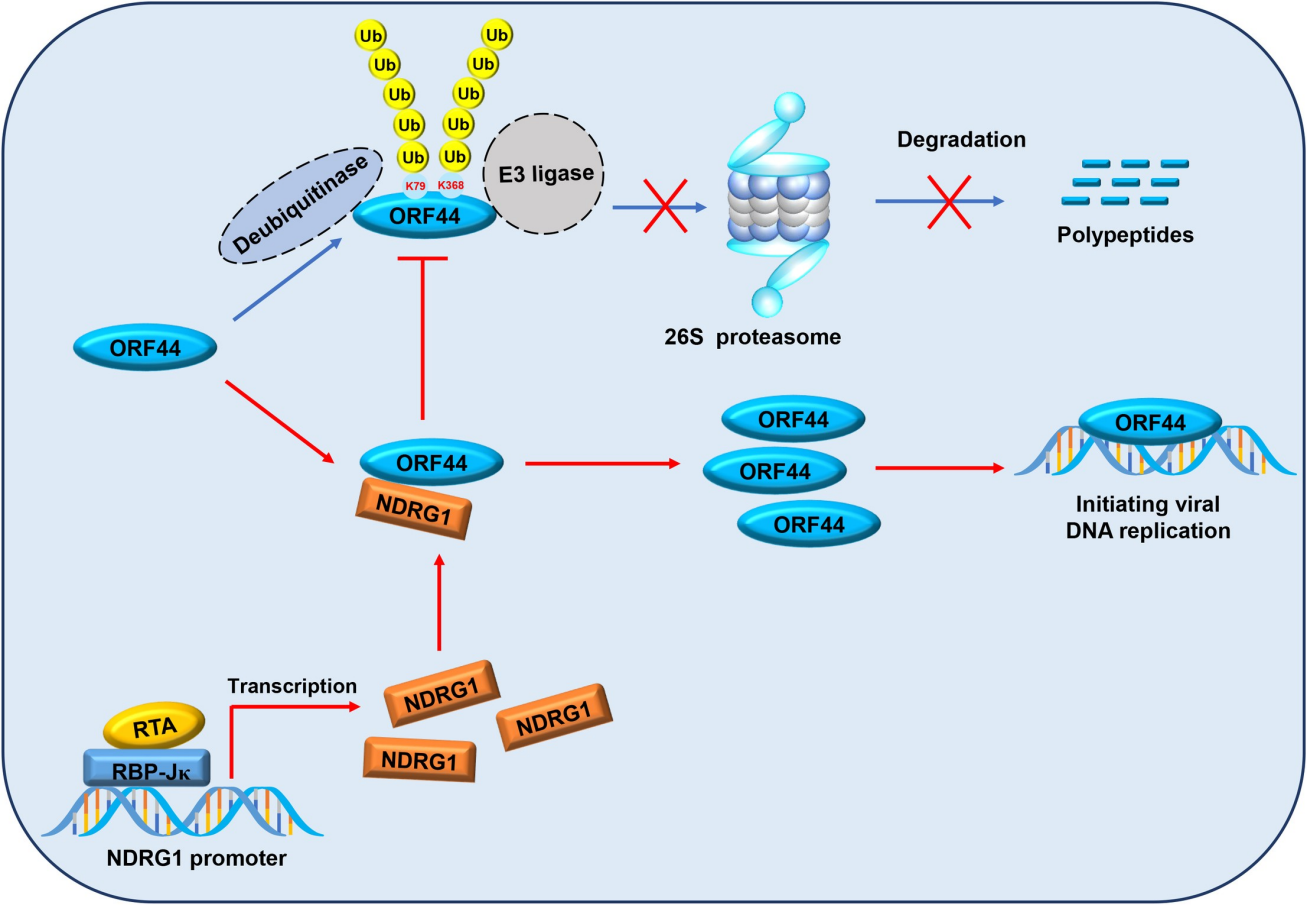
The working model for the function of NDRG1 in maintaining the stability of ORF44. During KSHV lytic replication, host protein NDRG1 is highly upregulated by RTA and cofactor RBP-Jκ. NDRG1 further interacts with KSHV ORF44 and impairs the polyubiquitination of lysine residues at positions 79 and 368 in ORF44 to inhibit its ubiquitin-proteasome-mediated degradation. The rescued ORF44 binds to KSHV genome and performs the function of unwinding viral double-stranded DNA and initiating DNA replication.

Previous studies have shown that helicases play a central role in DNA replication, damage signaling, and repair; telomere maintenance; and genome stability. Recent studies have identified posttranslational modifications (PTMs) of helicases, such as phosphorylation, ubiquitination, small ubiquitin-like modifier (SUMO)ylation, O-N-acetyl-D-glucosamine (GlcNAc)ylation and acetylation, which in turn regulate the protein-protein interactions, subcellular localization and protein stability of helicases [46–51]. In our previous study, we found that viperin catalyzes methionine oxidation of DNA and RNA helicases, which enhances their stability and enzyme activity to promote their functions. In this study, we found that RTA and the cofactor RBP-Jκ significantly upregulates the host protein NDRG1 to interact with ORF44 and that NDRG1 inhibits ubiquitin-proteasome-mediated degradation of ORF44 by impairing its polyubiquitination at K79 and K368 to facilitate viral lytic replication. These findings demonstrate that NDRG1 is a positive regulator of ORF44 expression and facilitates KSHV lytic replication. In summary, our results establish a working model illustrating the function of NDRG1 in maintaining the protein stability of ORF44 (Fig 8).

Ubiquitination is a reversible posttranslational modification that regulates various cellular processes, such as protein stability, binding interactions, DNA replication, cell cycle progression, cellular metabolism and innate immune responses [44,52–54]. Ubiquitin is a 76-amino-acid small peptide (8.6 kDa), and all seven lysine residues and the first N-terminal methionine in ubiquitin can covalently conjugate with one or more lysine residues in the substrate protein, forming a diverse combination of mono- or polyubiquitinated proteins with linear or branched ubiquitin chains. Ubiquitin modification requires a set of enzymatic reactions: activation by an E1 activating enzyme, conjugation by an E2 ubiquitin-conjugating enzyme, and ligation by an E3 ubiquitin ligase [45,50,55,56]. To date, numerous studies have shown that the human genome encodes 2 E1 activating enzymes, approximately 40 E2 ubiquitin-conjugating enzymes and over 600 E3 ubiquitin ligases [57]. In addition, some viruses can hijack and manipulate the ubiquitin system to overcome host defenses and control viral infection, uncoating, genome replication and egress [58–62]. Our present study showed that KSHV also strongly upregulates NDRG1 expression through RTA and that NDRG1 interacts with ORF44 to inhibit its ubiquitin-proteasome-mediated degradation, thus facilitating KSHV lytic replication. However, the specific molecular mechanism of ORF44 deubiquitination has not been fully elucidated. On the one hand, NDRG1 is not an E3 ubiquitin ligase and we speculate that NDRG1 may compete with certain E3 ubiquitin ligases for binding to the same region of ORF44, impairing its degradation. Next, we intend to further investigate the E3 ubiquitin ligases interacting with ORF44 through tandem affinity purification/mass spectrometry detection and bioinformatic analysis. On the other hand, some groups have reported that deubiquitinases (DUBs) can remove ubiquitin from substrate proteins. The human genome encodes approximately 100 deubiquitinases, which are classified into seven families based on their evolutionary conservation [63,64]. Combining these findings, we propose another hypothesis that NDRG1, as a scaffold protein, interacts with ORF44 and recruits specific deubiquitinases to mediate ORF44 deubiquitination. Thus, we intend to perform related experiments to further search for deubiquitinases that may bind to ORF44 or NDRG1. In general, exploring the regulatory mechanisms of ORF44 ubiquitination and deubiquitination is essential for understanding KSHV lytic replication; thus, more investigation and discovery is required.

NDRG1, a well-established metastasis suppressor, is regulated by multiple biological stimuli, including heavy metal ions, hypoxia, free radical nitric oxide (•NO) and androgen [33,65,66]. In various types of cancers, such as prostate, pancreatic and colon cancers, NDRG1 plays an important role in inhibiting epithelial-mesenchymal transition (EMT), cell migration and angiogenesis [23,67,68]. However, NDRG1 also plays an oncogenic role in certain cancer types, including scirrhous gastric cancer and hepatocellular carcinoma, indicating that it performs pleiotropic functions depending on the cancer type [69,70]. Thus, appropriate upregulation or downregulation of NDRG1 expression has been proposed as a potent anticancer strategy. In PC3 prostate cancer cells, valproic acid (VPA), a clinically available histone deacetylase inhibitor, upregulates NDRG1 expression to inhibit cancer cell invasion [71]. Similarly, in PANC-1 pancreatic cancer cells, trichostatin A (TSA), another histone deacetylase inhibitor, highly upregulates the mRNA and protein expression of NDRG1 to induce differentiation [72]. Further, extensive studies have indicated that NDRG1 is upregulated by iron ions in various cancer cells, resulting in its potent anticancer activity [68,73]. On the other hand, abrogation of NDRG1 expression can sensitize osteosarcoma cells to combination therapy with combretastatin A-4 and chloroquine via suppression of autophagosome-lysosome fusion followed by promotion of apoptosis [74]. In addition, NDRG1 downregulation sensitizes radiotherapy-resistant human rectal cancer cells to radiation by increasing DNA double-strand breaks [75]. These findings provide additional insight into NDRG1 as a promising therapeutic target for anticancer strategies. Moreover, both our previous studies and our present study demonstrated that NDRG1 is highly upregulated during both latency and lytic replication and plays a crucial role in KSHV genomic persistence and viral lytic replication. Considering the close relationship between NDRG1 and the life cycle of KSHV, we also believe that NDRG1 may be a therapeutic target for KSHV-related diseases. Next, we intend to screen small molecule compounds and design small peptides to regulate NDRG1 expression, which further impairs the life cycle of KSHV, to treat related diseases.

In addition, the NDRG1-Flag reconstituted samples in Fig 5F showed weaker viral phenotypes to WT samples in Fig 5C, which might be caused by the different experimental methods of using the transient lentivirus infection rather than isolating the positive monoclones. Moreover, the relevant experiment results showed that the restoration of ORF44 expression in NDRG1-deficient iSLK.RGB cells dramatically increased both intracellular and extracellular viral genome copy numbers. Although the restoration of ORF44 expression in NDRG1-deficient iSLK.RGB cells showed weaker viral phenotypes to the restoration of NDRG1 expression, but they appeared similar in magnitude. These results showed that NDRG1 is essential for KSHV lytic replication, indicating that NDRG1 mainly relied on ORF44 to facilitate viral lytic replication. Moreover, it also suggested that NDRG1 might contribute to KSHV lytic replication through other molecular mechanisms. Therefore, we need to further investigate.

In conclusion, our work showed for the first time that ORF44 is a novel NDRG1-binding protein and that NDRG1 maintains the stability of ORF44 by inhibiting its ubiquitin-proteasome-mediated degradation to promote KSHV lytic replication. Because of the crucial role of NDRG1 in both the latency and lytic replication of KSHV, NDRG1 downregulation in KSHV-positive cells may be an effective therapeutic and prophylactic strategy for KSHV-related diseases.

## Materials and methods

### Cell culture

The HEK293T, Hela, iSLK.RGB and iSLK-BAC16 KSHV helicase-Flag (RGB deletion) cell lines were maintained in high-glucose Dulbecco’s modified Eagle’s medium (DMEM, Biological Industries) supplemented with 10% fetal bovine serum (FBS, Biological Industries), 1% antibiotics (penicillin and streptomycin, Gibco), and the appropriate selective pressures (puromycin,1.5 μg/ml; G418, 0.5 mg/ml; hygromycin, 0.5 mg/ml). All the cell lines were cultured at 37°C in the presence of 5% CO2. The iSLK.RGB cell line was kind gifts from Dr. Jae Jung (University of Southern California) and Dr. Fanxiu Zhu (Florida State University). The HEK293T and Hela cell lines were from our laboratory stock. The iSLK-BAC16 KSHV helicase-FLAG (GFP deletion) cell line was described previously [22].

### Antibodies and reagents

The following primary antibodies were used: anti-NDRG1 rabbit monoclonal antibody (Abcam, ab124689), anti-NDRG1 mouse monoclonal antibody (Sigma, AP1160), anti-UBC rabbit polyclonal antibody (ABclonal, A3207), anti-Rabbit Control IgG antibody (ABclonal, AC005), anti-GAPDH mouse monoclonal antibody (ABclonal, AC033), anti-β-actin rabbit monoclonal antibody (ABclonal, AC026), anti-α-tubulin mouse monoclonal antibody (Sigma, T6199), anti-Flag antibody (Sigma, F7425 and F1804), anti-HA antibody (Sigma, H9658 and H6908), anti-LANA rat monoclonal antibody (Advanced Biotechnology Inc, 13-210-1000), anti-ORF45 and ORF65 mouse monoclonal antibody (a kind gift from Yan Yuan, University of Pennsylvania, Philadelphia, Pennsylvania, USA), and anti-RTA rabbit monoclonal antibody were prepared in our laboratory. The secondary antibodies were as follows: goat anti-mouse IgG light chain (Jackson ImmunoResearch Laboratories, 115-035-174), goat anti-rabbit IgG (H+L) (Jackson ImmunoResearch Laboratories, 111-005-144), goat anti-mouse antibodies conjugated with Alexa Fluor 488 (Invitrogen, A-11001) and 555 (Invitrogen, A-21422), goat anti-rabbit antibodies conjugated with Alexa Fluor 488 (Invitrogen, A-11094) and 555 (Invitrogen, A-27017). The other reagents used and their sources were as follows: recombinant protein A agarose (Invitrogen, 15948–014), recombinant protein G agarose (Invitrogen, 15920–010), anti-Flag M2 affinity gel (Sigma, A2220), Lipofectamine 2000 (ThermoFisher Scientific, 11668019), MG132 (MedChemExpress, HY-13259), cycloheximide (CHX) (MedChemExpress, HY-12320), 3-Methyladenine (3MA) (MedChemExpress, HY-19312), chloroquine (MedChemExpress, HY-17589A), doxycycline hyclate (Sigma, 324385), blasticidin (Sigma, 203350), protease inhibitor cocktail (Sigma, P8340).

### Plasmids

The Full-length fragment of NDRG1 was amplified from a iSLK.RGB cDNA library, inserted into Streptomycin-Flag-tagged pCDH and HA-tagged pCMV vectors. The KSHV ORF44, RTA expression plasmids were previously described [22,76]. Mammalian expression plasmids for ORF44 and NDRG1 truncations were constructed by standard molecular biology techniques (see the schematics in Fig 2A, 2C and 2E). The ORF44 mutant plasmids were generated by using the pCMV-HA-ORF44 plasmid as a template following the manufacturer’s protocol of the Fast Site-Directed Mutagenesis Kit (TIANGEN) (see the schematics in Fig 7A). The luciferase reporter plasmid pGL3-Basic-pNDRG1 was constructed by cloning the promoter regions of NDRG1 (-2000 to -1 bp) from the iSLK.RGB genomic library into the pGL3-Basic vector. The reporter plasmid pGL3-Basic-pNDRG1-ΔRBP-Jκ was constructed by using the pGL3-Basic-pNDRG1 plasmid as a template and the Fast Site-Directed Mutagenesis Kit (TIANGEN) (see the schematics in Fig 3C). All the primers used for gene amplification are listed in S2 Table.

### Coimmunoprecipitation (co-IP) and Immunoblotting

Cells were lysed in RIPA buffer (Beyotime, P0013D) supplemented with protease inhibitor cocktail and 1mM PMSF for 1 h on Rotational Incubator (Kylin-Bell Lab Instruments, QB128) at 4°C. Then the cells were centrifuged at 12,000 rpm for 15 min at 4°C to remove cell debris. Five percent of the cell lysates were taken as the input, and the remainder was immunoprecipitated with affinity beads or the corresponding antibodies overnight at 4°C. The immunoprecipitate was washed three times with RIPA buffer and boiled in SDS loading buffer at 100°C for 10 minutes. For immunoblotting analysis, the treated protein samples were analyzed by SDS-PAGE and transferred to nitrocellulose membranes, followed by blocking and probing with the indicated antibodies for detection.

### Immunofluorescence assay

Cells were washed thrice with PBS and fixed with 4% paraformaldehyde (Beijing Dingguo Changsheng Biotechnology) for 30 min at room temperature. Then cells were permeabilized with 0.2% Triton X-100 for 10 min and blocked with 5% bovine serum albumin (Life Technologies) in PBS for 1 h, followed by incubation with the indicated antibodies (1:200 dilution) overnight at 4°C and further staining with the secondary antibodies (1:400 dilution) for 1 h at room temperature. Cell nuclei were stained with DAPI (Beyotime, C1002) for 5 min. Finally, slides were photographed by a digital camera and software (Leica, SP8).

### Quantitative real-time PCR (RT-qPCR)

Cells were harvested and lysed in the TRIzol reagent (Invitrogen), then the total RNA was extracted according to the manufacturer’s instructions. Three microgram of RNA was reverse transcribed into cDNA with HiScript III RT SuperMix (Vazyme, R323-01). RT-qPCR was performed with a Hieff qPCR SYBR Green Master Mix (YEASEN, 11202ES03) on QuantStudio 6 Flex Real-Time PCR System (Applied Biosystems) according to the manufacturer’s instructions. Relative mRNA levels were normalized to GAPDH and calculated via the ΔΔCT method. To analyze viral genomic DNA level, intracellular viral genomic DNA and extracellular virion DNA were extracted from induced cells or the cell supernatants with the Genomic DNA Extraction Kit (TIANGEN). The KSHV genomic DNA copy numbers were quantified by RT-qPCR using primers K9. The samples were tested in triplicate. The primers used in RT-qPCR are listed in S2 Table.

### Dual-luciferase reporter assay

A dual-luciferase reporter assay system (Promega) was used according to the manufacturer’s instructions. The expressing Renilla luciferase plasmids pRL-TK were used to normalize firefly luciferase activity. The day before transfection, HEK293T cells were seeded into 12-well plates at appropriate density, then the cells were cotransfected with the indicated luciferase reporter plasmids and the Flag-tagged RTA plasmid or empty vector plasmid. At 24~48 h post-transfection, cells were harvested and lysed in 200μl cell lysis buffer to detect luciferase activity.

### RNA interference

iSLK.RGB cells were transfected with negative control siRNA or siRNAs corresponding to the indicated genes (GenePharma Technology) using Lipofectamine 2000 according to the manufacturer’s instructions. At 24~48 h post-transfection, the cells were harvested and the efficiency of RNA interference was detected by immunoblotting analysis. All siRNA used in our experiments and their sequences are as follows:

negative control siRNA, 5’-UUCUCCGAACGUGUCACGUTT-3’;
siRTA#1, 5’-GUGCCGUGUAGAGAUUCAATT-3’;
siRTA#2, 5’-GUACCUCUUUGGGAUCAAUTT-3’;
siNDRG1#1, 5’-CAUCGAGACUUUACAUGGCUCUGUU-3’;
siNDRG1#2, 5’-GCUGAUCCAGUUUCCGGAATT-3’;
siViperin#1, 5’-CAACCAGCGUCAACUAUCATT-3’;
siViperin#2, 5’-GGUGUAGGGAUUAUAGAGUTT-3’.

### Infection of 293T cells with KSHV progeny virus

The day before infection, HEK293T cells were seeded into 12-well plates at appropriate density. The cells in each well were incubated with 1ml cell culture supernatants, then the plates were centrifuged at 2500 rpm for 2 h at 37°C. The incubated supernatants were replaced with fresh medium, and the cells were cultured for 24 h. The virus infection rate of 293T cells was detected by fluorescence microscopy according to RFP signal intensity.

### Constitution of NDRG1-deficient iSLK.RGB cell line

NDRG1-deficient iSLK.RGB cell line was constructed through the lentiviral CRISPR/Cas9 system as described previously [77]. The sequence of a single guide RNA (sgRNA) targeting human NDRG1 gene was cloned into lentiCas9-Blast vector, then the indicated plasmid was cotransfected into HEK293T cells with the packaging plasmids to produce the recombined lentivirus. iSLK.RGB cells were centrifuged with empty vector lentiCas9-Blast lentivirus (negative control) or NDRG1 lentiCas9-Blast lentivirus at 2500 rpm for 2 h at 37°C. At 48 h post-infection, the cells were added blasticidin (25 μg/ml) to select the positive clones. The monoclonal cells can be screened out by limited dilution method after more than one month, and the effect of NDRG1 knockout was confirmed by immunoblotting analysis. All sgRNAs used and their sequences are as follows:

sgNDRG1#1, 5’- CACAAGGGTTCACGTTGATA -3’;
sgNDRG1#2, 5’- GGGTTCACGTTGATAAGGAC-3’;
sgNDRG1#3, 5’- CTCTTCAACTACGAGGACAT -3’.

### Cycloheximide chase assay

HEK293T cells were cotransfected with the indicated plasmids. After 24 h post-transfection, the cells were treated with 20 μM CHX to inhibit protein synthesis. At the indicated time points, the cells were harvested and lysed for immunoblotting analysis. The intensity of each ORF44 band was first normalized with the intensity of its corresponding tubulin, followed by comparing to the normalized ORF44 value at 0 h, and data was analyzed by GraphPad Prism software.

### Ubiquitination assay

First, HEK293T cells were seeded into 6-well plates at appropriate density. The cells were cotransfected with indicated plasmids and then treated with MG132 (20 μM) or DMSO for 12 h. After 48 h post-transfection, the cells were lysed and immunoprecipitated with anti-HA antibody, followed by the immunoprecipitates were analyzed by immunoblotting using anti-UBC rabbit polyclonal antibody.

### Statistical analysis

The statistical analysis was performed through GraphPad Prism software. Data were determined by the unpaired, two tailed Student’s t-tests and statistical significance was set as P-value < 0.05. Error bars represent as mean ± SD. Each experiment was carried out independently at least three times.

## Supporting information

S1 Fig Silencing RTA reduces NDRG1 expression during viral lytic replication. (A) iSLK.RGB cells were transfected with siRNA as indicated. At 24 h post-transfection, the cells were treated with doxycycline for 48 h. The knockdown efficiency of RTA was determined by immunoblotting (left panel) and qPCR analysis (right panel). (B) iSLK.RGB cells were transfected with indicated siRNA for 24 h. Then, the cells were induced by doxycycline. The expression of RTA and NDRG1 at indicated time points were detected by immunoblotting (left panel) and qPCR analysis (right panel). Data were shown as mean ± SD, n = 3; ns, not significant; ***p <0.001; ****p <0.0001.
(TIF)

S2 Fig NDRG1-deficient iSLK.RGB cell clones exhibit consistent viral phenotypes. (A and B) The knockout efficiency of NDRG1 in DRG1-deficient iSLK.RGB cell clones were confirmed by immunoblotting and Sanger sequencing. (C and D) Wild-type iSLK.RGB cell clones and NDRG1-deficient iSLK.RGB cell clones were treated with doxycycline, then intracellular viral genomic DNA (C) and extracellular virion DNA (D) were extracted from the induced cells or cells supernatants. The KSHV genomic DNA copy numbers at indicated time points were detected by qPCR analysis. Data were shown as mean ± SD, n = 3; **p < 0.01; ***p < 0.001; ****p <0.0001.
(TIF)

S3 Fig NDRG1 increases ORF44 stability independent of Viperin. (A) HEK293T cells were transfected with siRNA as indicated for 48 h. The knockdown efficiency of Viperin was determined by immunoblotting (left panel) and qPCR analysis (right panel). (B) HEK293T cells were transfected with indicated siRNA for 24 h, then the cells were cotransfected with plasmids encoding HA-tagged ORF44 and Flag-tagged NDRG1 or empty vector for 48 h. The protein expression levels of ORF44, NDRG1 and Viperin were detected by immunoblotting analysis. Data were shown as mean ± SD, n = 3; *p < 0.05; **p < 0.01.
(TIF)

S4 Fig The protein abundance of ORF44 single lysine mutants. (A) Schematic diagram of the positions of all 34 lysine residues in ORF44. According to the lysine residue position, ORF44 is divided into five clusters, including A6, B6, C7, D6 and E9. (B-F) HEK293T cells were transfected with the indicated plasmids for 48 h, then the cells lysed and the protein abundance of these mutants were detected by immunoblotting.
(TIF)

S1 Table NDRG1 interacted with KSHV-encoded proteins identified in TAP-MS. (XLSX)

S2 Table Primers for PCR amplification and analysis. (XLSX)
