# Supplementary figures and images for: NDRG1 facilitates lytic replication of Kaposi’s sarcoma-associated herpesvirus by maintaining the stability of the KSHV helicase
Source: PLoS Pathog. 2021 Jun 2;17(6):e1009645. doi: 10.1371/journal.ppat.1009645 (PMC8202935; doi:10.1371/journal.ppat.1009645)

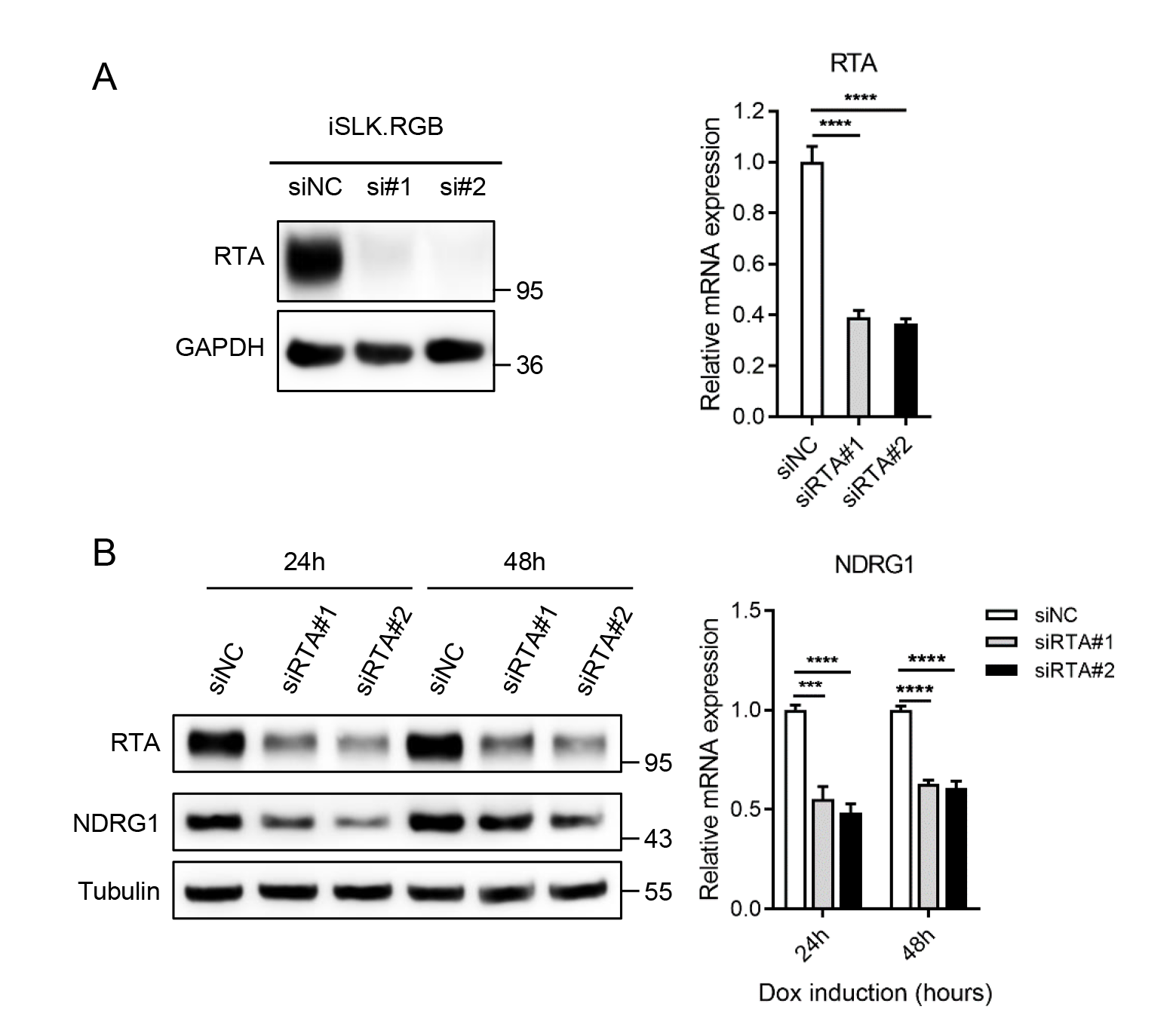

Supplement: S1 Fig — (A) iSLK.RGB cells were transfected with siRNA as indicated. At 24 h post-transfection, the cells were treated with doxycycline for 48 h. The knockdown efficiency of RTA was determined by immunoblotting (left panel) and qPCR analysis (right panel). (B) iSLK.RGB cells were transfected with indicated siRNA for 24 h. Then, the cells were induced by doxycycline. The expression of RTA and NDRG1 at indicated time points were detected by immunoblotting (left panel) and qPCR analysis (right panel). Data were shown as mean ± SD, n = 3; ns, not significant; ***p <0.001; ****p <0.0001. (TIF) [file ppat.1009645.s001.tif]

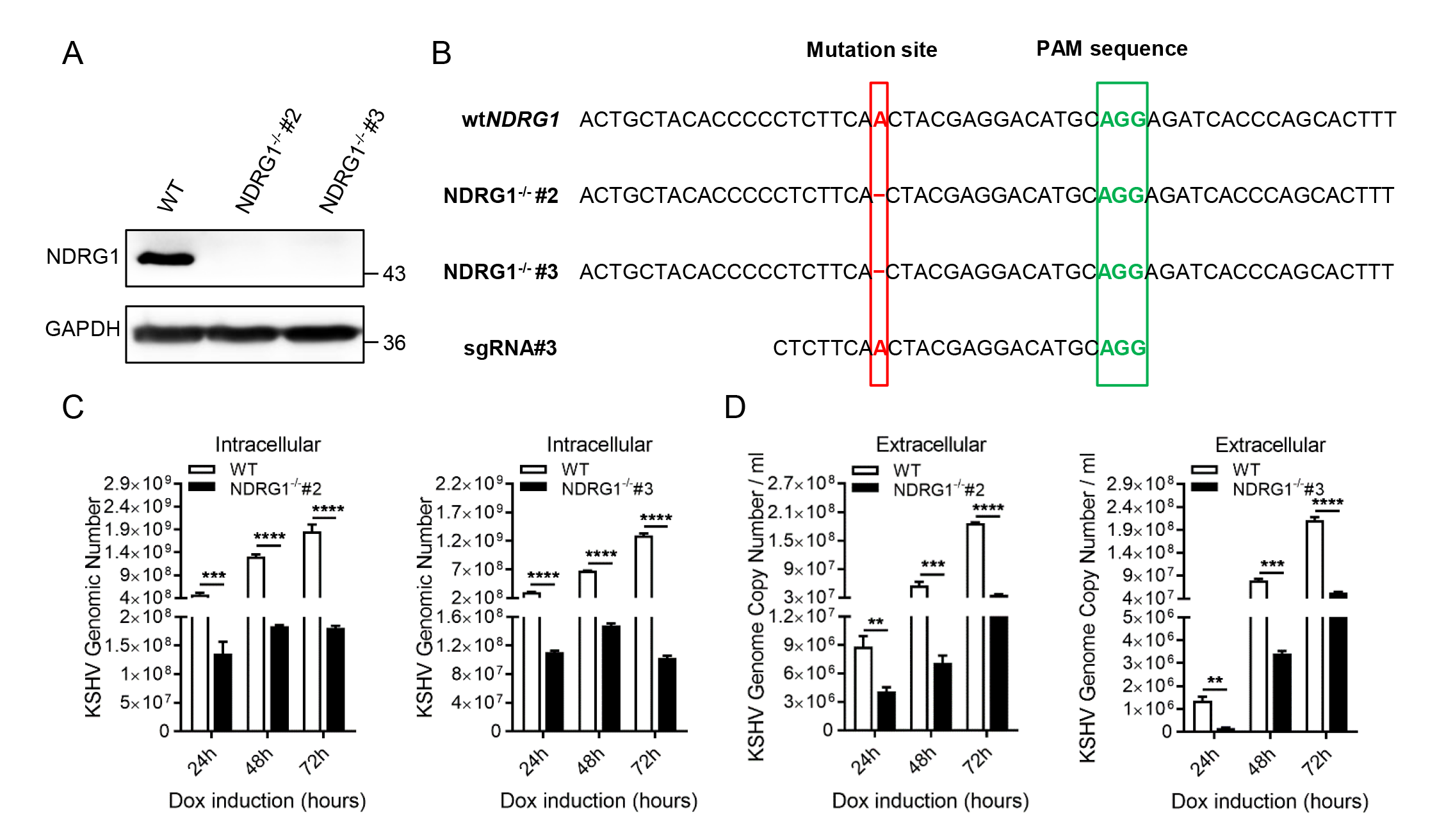

Supplement: S2 Fig — (A and B) The knockout efficiency of NDRG1 in DRG1-deficient iSLK.RGB cell clones were confirmed by immunoblotting and Sanger sequencing. (C and D) Wild-type iSLK.RGB cell clones and NDRG1-deficient iSLK.RGB cell clones were treated with doxycycline, then intracellular viral genomic DNA (C) and extracellular virion DNA (D) were extracted from the induced cells or cells supernatants. The KSHV genomic DNA copy numbers at indicated time points were detected by qPCR analysis. Data were shown as mean ± SD, n = 3; **p < 0.01; ***p < 0.001; ****p <0.0001. (TIF) [file ppat.1009645.s002.tif]

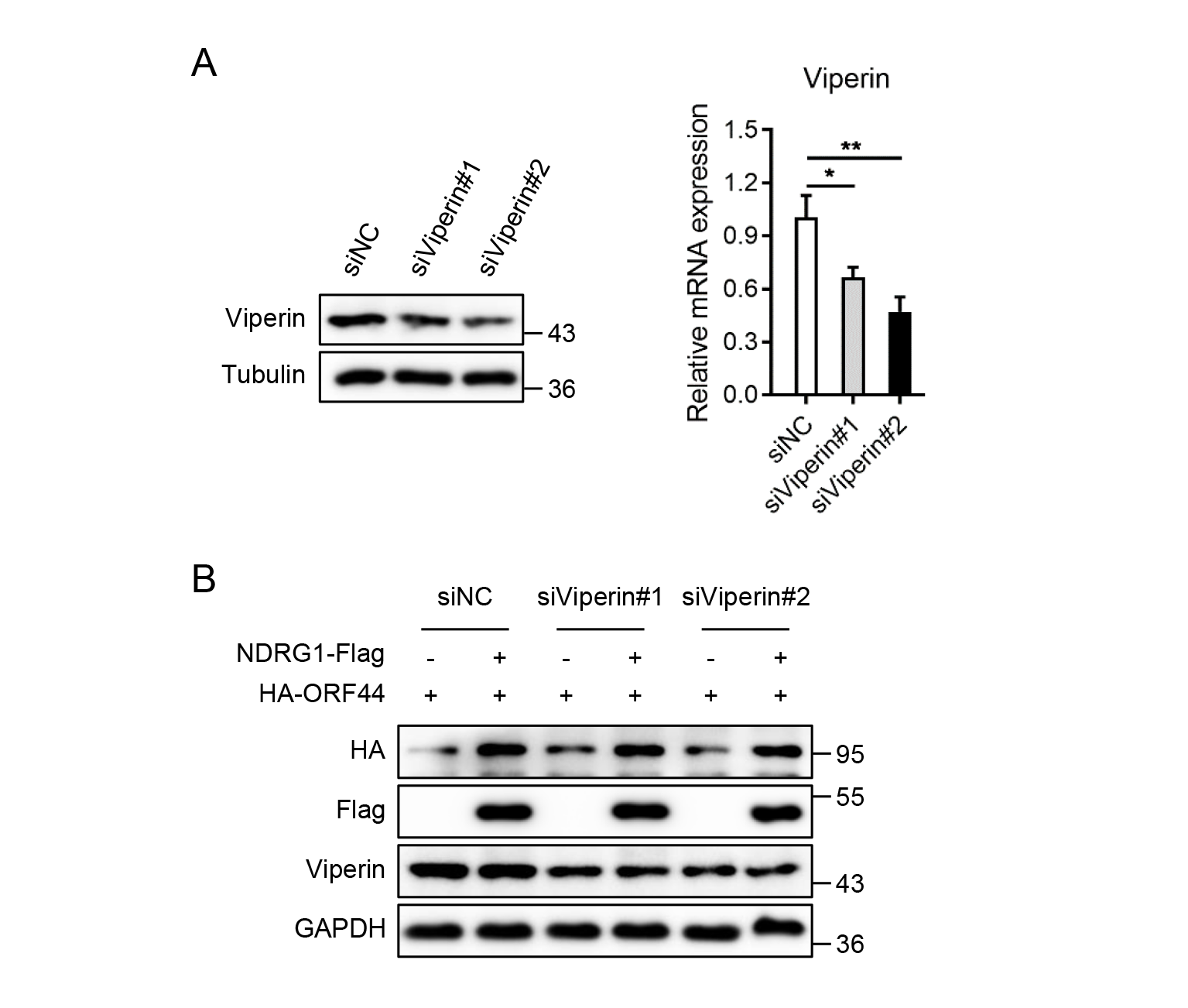

Supplement: S3 Fig — (A) HEK293T cells were transfected with siRNA as indicated for 48 h. The knockdown efficiency of Viperin was determined by immunoblotting (left panel) and qPCR analysis (right panel). (B) HEK293T cells were transfected with indicated siRNA for 24 h, then the cells were cotransfected with plasmids encoding HA-tagged ORF44 and Flag-tagged NDRG1 or empty vector for 48 h. The protein expression levels of ORF44, NDRG1 and Viperin were detected by immunoblotting analysis. Data were shown as mean ± SD, n = 3; *p < 0.05; **p < 0.01. (TIF) [file ppat.1009645.s003.tif]

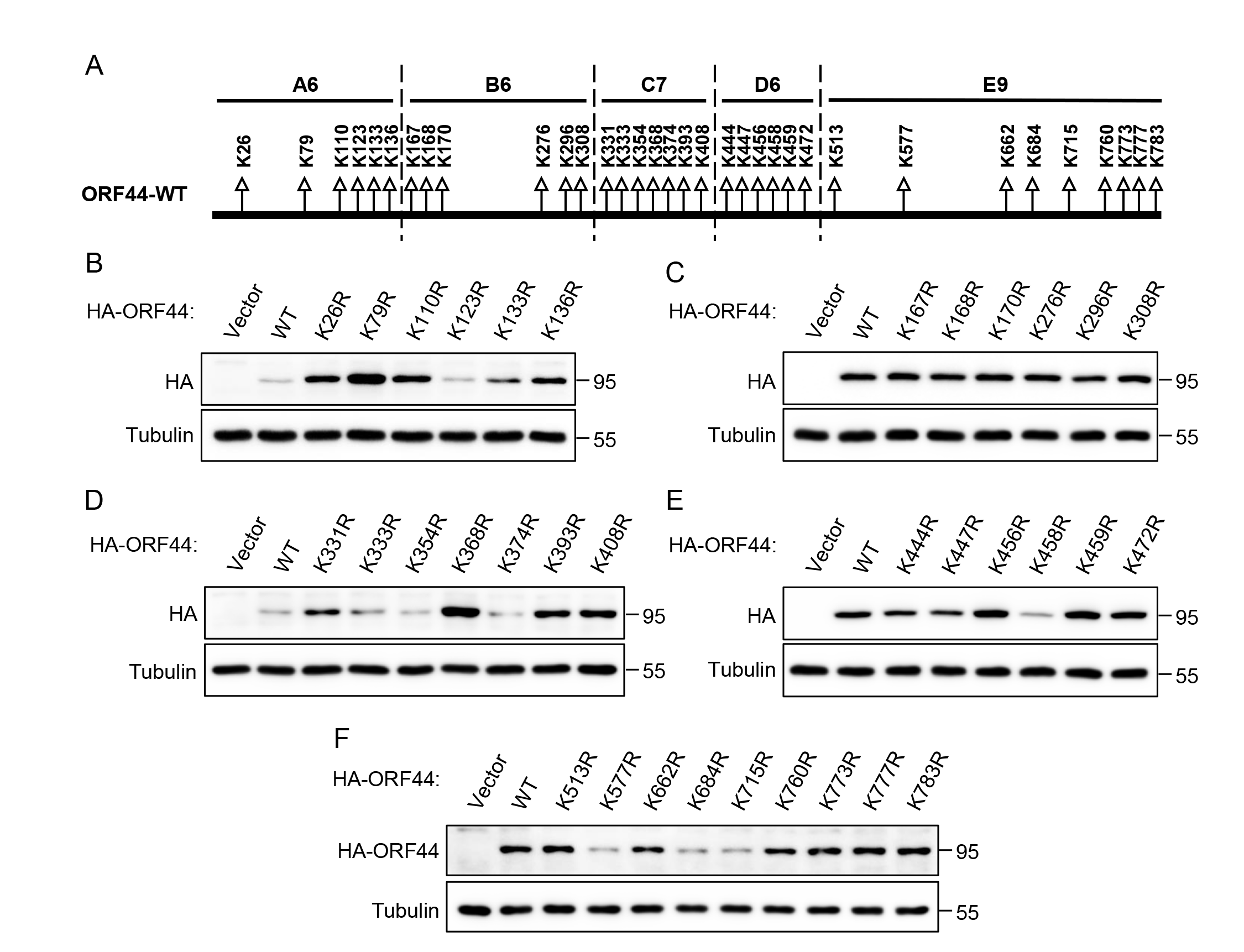

Supplement: S4 Fig — (A) Schematic diagram of the positions of all 34 lysine residues in ORF44. According to the lysine residue position, ORF44 is divided into five clusters, including A6, B6, C7, D6 and E9. (B-F) HEK293T cells were transfected with the indicated plasmids for 48 h, then the cells lysed and the protein abundance of these mutants were detected by immunoblotting. (TIF) [file ppat.1009645.s004.tif]
